# Osteocytes orchestrate browning: emerging signals in bone-fat crosstalk: a systematic review

**DOI:** 10.3389/fendo.2026.1766959

**Published:** 2026-03-06

**Authors:** Houmam Anees, Vahid Jahed, Zahra Sabouri, Reem Jamous, Cristian Pablo Pennisi, Christian Heiss, Thaqif El Khassawna

**Affiliations:** 1 Department of Trauma, Hand and Reconstructive Surgery, Faculty of Medicine, Justus-Liebig-University of Giessen, Giessen, Germany; 2 Experimental Trauma Surgery, Faculty of Medicine, Justus-Liebig-University of Giessen, Giessen, Germany; 3 Regenerative Medicine, Department of Health Science and Technology, Faculty of Medicine, Aalborg University, Aalborg, Denmark; 4 Biruni University, Istanbul, Türkiye; 5 School of Pharmacy, The University of Jordan, Amman, Jordan

**Keywords:** adipocyte browning, bone–fat crosstalk, energy metabolism, osteocytes, sclerostin (SOST), thermogenesis

## Abstract

**Background:**

Bone is increasingly viewed as an active component of systemic physiology rather than a solely structural tissue. Among its resident cells, osteocytes have emerged as important endocrine regulators capable of influencing metabolic processes in distant organs. Several osteocyte-derived factors have been linked to pathways that govern energy balance. Recent work suggests that these signals may also affect the browning of white adipose tissue, a thermogenic remodeling process.

**Scope of review:**

This systematic review specifically examines the evidence supporting the regulatory role of osteocytes in adipocyte browning. The experimental studies investigated how osteocyte-related signals—such as sclerostin suppression, PPARγ deletion, Sirt1 activation, or downstream BMP modulation—affect thermogenic gene expression, and beige adipocyte differentiation across peripheral and marrow fat depots. Despite heterogeneity in design and endpoints, the collectively available evidence indicates that osteocytes can influence the induction or repression of adipocyte browning programs.

**Major conclusion:**

Current evidence supports a role for osteocytes as modulators of adipocyte browning, integrating skeletal signaling with systemic metabolic responses. However, the findings remain preliminary due to limited osteocyte-specific targeting, depot-specific variability, and contextual differences across models. Establishing the physiological relevance of osteocyte-derived signals and defining the conditions under which they influence adipose plasticity will be essential for advancing therapeutic exploration. Understanding this bone–fat crosstalk may ultimately provide new opportunities to address metabolic and skeletal disorders through shared regulatory pathways.

## Highlights

- Osteocytes influence adipocyte browning by secreting extracellular signals (e.g., sclerostin, BMPs) and modulating intracellular regulators (e.g., Sirt1 promotes thermogenesis; PPARγ favors adipogenesis), thus linking bone remodeling to systemic energy metabolism.
- The effects of osteocyte-derived sclerostin on thermogenesis vary depending on fat depot, intervention type, and systemic context, reflecting a complex regulatory network.
- Evidence from murine and human models supports the concept that bone-derived signals influence adipocyte phenotype, offering new insights into bone–fat crosstalk.
- Exercise, mechanical loading, and NAD^+^-dependent deacetylation link redox signaling to both bone remodeling and thermogenesis, suggesting shared oxidative control mechanisms.

## 1 Introduction

Traditionally viewed as a passive structure, bone has recently emerged as a dynamic endocrine organ influencing whole-body metabolism. Osteocytes, the most abundant bone cells, are increasingly recognized for their role in systemic regulation through secreted signals such as sclerostin (SOST), Peroxisome proliferator-activated receptor gamma (PPARγ), Sirtuin 1 (Sirt1), and Bone morphogenetic protein 7 (BMP7) (1–4). These molecules not only control bone remodeling but also influence energy balance and fat metabolism (5–7). This bone–fat communication is particularly relevant in aging, osteoporosis, and metabolic disorders, where alterations in bone mass frequently coincide with changes in adipose tissue distribution and function (8, 9).

**Figure 1:**
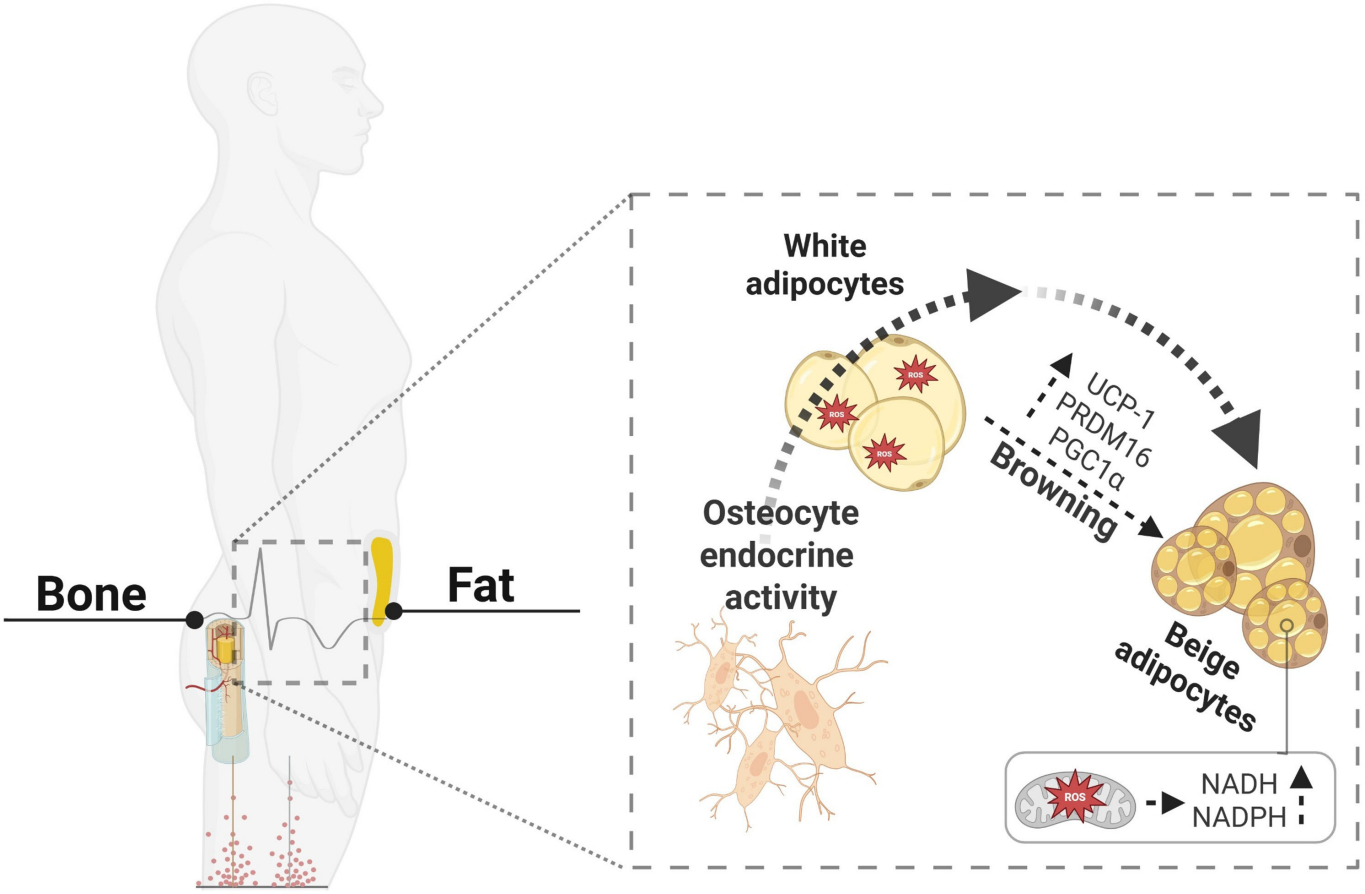
Osteocyte endocrine signaling links bone–fat communication. Schematic representation of the endocrine crosstalk between bone and adipose tissue. Osteocytes secrete key regulatory molecules that influence adipocyte phenotype and promote the browning of white adipocytes through activation of thermogenic genes (UCP1, PGC1α, PRDM16). This transition enhances mitochondrial biogenesis, increases NADH/NADPH-dependent oxidative metabolism, and improves reactive oxygen species (ROS) handling. In turn, beige adipocytes display superior antioxidant capacity and contribute to systemic redox homeostasis and energy expenditure. Created with BioRender.com.

White adipose tissue (WAT) stores energy, whereas brown adipose tissue (BAT) dissipates it through Uncoupling Protein 1 (UCP1)-mediated mitochondrial uncoupling. Under specific stimuli—including cold exposure, sympathetic activation, irisin, BMP7, and Fibroblast Growth Factor 21 (FGF21), white adipocytes can convert into thermogenic beige adipocytes (**Figure 1**), a process known as “browning” (10–15). Beige adipocytes display multilocular lipid droplets, high mitochondrial density, and upregulation of thermogenic genes such as UCP1 and Peroxisome Proliferator-Activated Receptor Gamma Coactivator 1-alpha (PGC1α), collectively enhancing oxidative metabolism and energy expenditure (16–18). This adaptive plasticity contributes to improved metabolic flexibility and protection against obesity and insulin resistance (10, 17).

Beyond its thermogenic role, browning represents a fundamental shift in redox physiology. White adipocytes possess limited antioxidant capacity and are prone to ROS accumulation and mitochondrial dysfunction under metabolic stress (19). In contrast, beige adipocytes exhibit enhanced mitochondrial ROS-buffering capacity and activate antioxidant pathways such as glutathione, thioredoxin, and pentose phosphate cycling (20, 21). Thus, the browning transition improves not only energy expenditure but also redox homeostasis. Osteocytes themselves are also redox-sensitive. Oxidative cues modulate signaling pathways such as Wnt/β-catenin and Sirt1, linking redox balance to osteocyte function and bone remodeling (22). These observations create a conceptual bridge whereby osteocyte-derived signals could influence adipocyte phenotypes through both endocrine and redox-dependent mechanisms.

Emerging evidence suggests that osteocyte-derived signals may directly influence adipocyte phenotypes. Sclerostin may promote beige adipogenesis via Wnt modulation and regulates adipocyte metabolic pathways (23). Sirt1 acts as a redox-sensitive deacetylase that suppresses the Sost gene (responsible for producing sclerostin), in osteocytes and promotes thermogenic gene expression in bone marrow adipocytes (24, 25). PPARγ in osteocytes influences systemic energy metabolism independently of circulating sclerostin (26), while BMP7 is a known inducer of brown/beige differentiation and mitochondrial biogenesis (27–29). Together, these pathways position osteocytes as potential upstream regulators of adipose browning (**Figure 1**).

Beyond classical UCP1 thermogenesis, adipocytes can generate heat through futile creatine, Ca²^+^, and lipid cycling, expanding the biochemical flexibility of thermogenic fat (10). In humans, browning is physiologically relevant: Sidossis et al. demonstrated that burn injury triggers multilocular UCP1^+^ adipocytes in subcutaneous WAT, establishing beige remodeling as a genuine adaptive response (12). Importantly, thermogenic adipocytes also act as secretory and endocrine cells, releasing so-called batokines, including FGF21, Interleukin-6 (IL-6), C-X-C Motif Chemokine Ligand 14 (CXCL14), and neuregulin-4 (NRG4), that exert autocrine, paracrine, and endocrine actions on liver, muscle, pancreas, and bone (11, 30). These mediators link adaptive thermogenesis to systemic glucose–lipid homeostasis. Nutrient-derived signals, such as TRP-channel agonists and dietary bioactives, can further potentiate browning responses and improve insulin sensitivity (31).

Despite these insights, the specific role of osteocytes in regulating adipocyte phenotypes remains underexplored. Most studies rely on murine models without osteocyte-specific targeting, and translational data are scarce. To date, no systematic review has comprehensively evaluated osteocyte-driven browning across adipose depots. This systematic review addresses that gap by integrating findings from five experimental studies alongside emerging translational observations to clarify how osteocytes influence energy metabolism.

## 2 Search strategy

We conducted comprehensive systematic search in PubMed, Embase, Web of Science, and Cochrane CENTRAL, aiming to identify original studies investigating the role of osteocytes or osteocyte-derived factors in the induction of adipocyte browning. It should be noted that bone marrow adipocytes represent a distinct adipocyte subtype with unique functional properties. Whether osteocyte-derived signals can influence browning-like features in bone marrow adipocytes remains an open question that was beyond the scope of the present study. The search covered all records up to 5 May 2025. Keywords and MeSH terms included combinations of:

“osteocyte”, “bone”, “sclerostin”, “Wnt”, “irisin”, “PPARγ”, “Gsα”, “Sirt1” with

“adipocyte”, “browning”, “beige adipose”, “thermogenesis”, “UCP1”, “white adipose”, “fat”, “energy metabolism”.

The full search strings for each database are provided in a **Supplementary File**.

### 2.1 Eligibility criteria

We included original animal, *in vitro*, and human studies that fulfilled the following criteria:

- Manipulated or measured osteocyte activity or osteocyte-derived factors
- Reported outcomes related to adipocyte browning or thermogenic gene expression
- Presented sufficient methodological detail and outcome data

We excluded:

- Reviews, editorials, conference abstracts without full data
- Studies investigating bone-fat interactions unrelated to browning
- Non-English language papers without translations

### 2.2 Study selection

**Figure 2:**
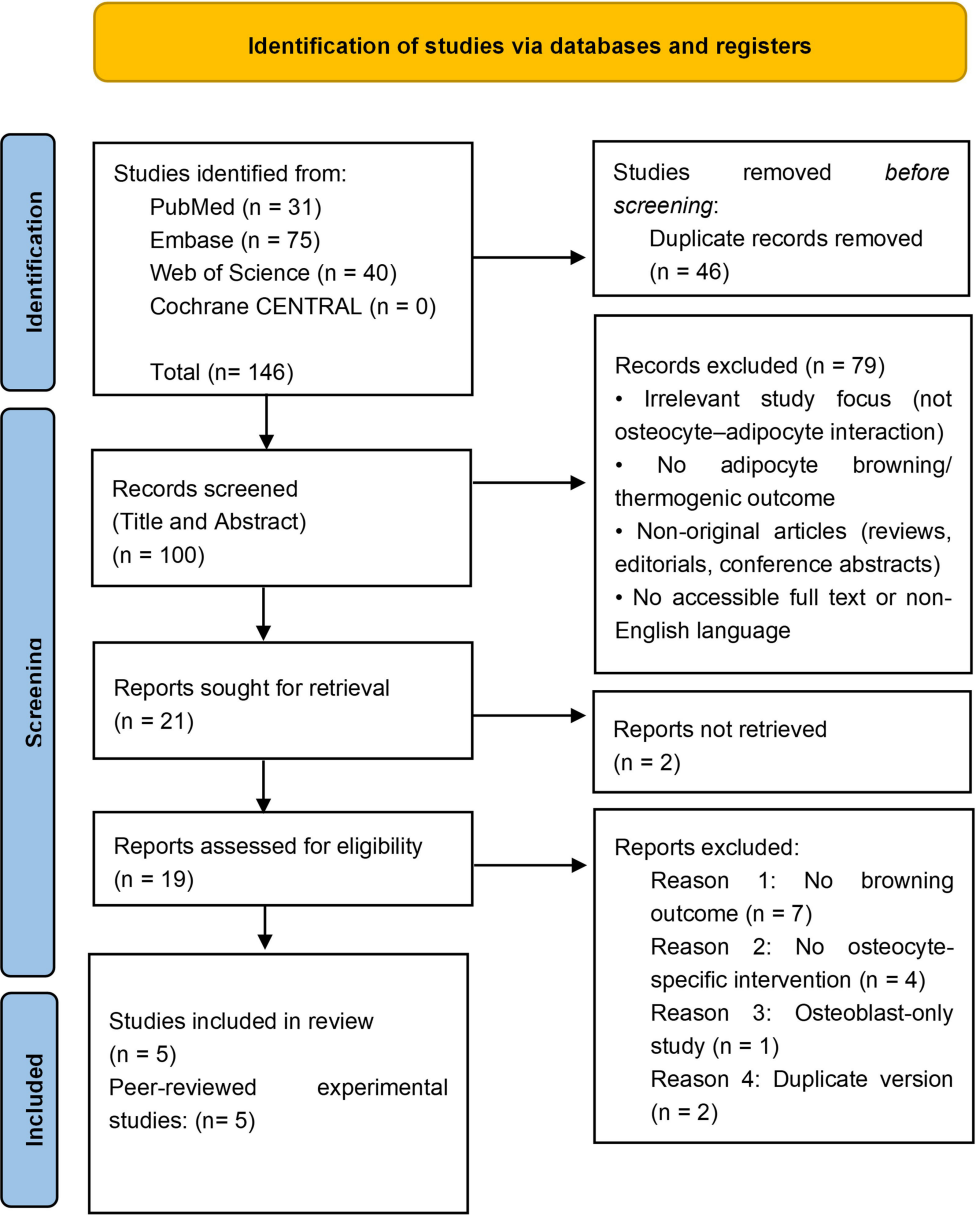
PRISMA flow diagram (32). n = number of records.

Titles and abstracts were screened in the first step. Full texts were assessed for inclusion based on the criteria above. Screening and eligibility assessment were performed independently by two reviewers, with discrepancies resolved by discussion and consensus. Screening results are summarized in the PRISMA flow diagram (**Figure 2**).

### 2.3 Risk of bias assessment

To assess internal validity of the animal studies, we used the SYRCLE Risk of Bias (RoB) tool (33), which includes 10 domains covering selection, performance, detection, attrition, and reporting biases. Each domain was rated as low, high, or unclear risk based on information reported in the full texts and supplementary files of the included studies. Ratings were made conservatively and in duplicate.

## 3 Results

Five preclinical studies published between 2017 and 2023 met the inclusion criteria for this review (**Table 1**). All used murine models, four incorporated *in vitro* assays, and one (Artsi et al.) included human-derived bone marrow stromal cells. These studies explored how osteocyte-associated pathways influence adipose phenotypes, with particular focus on thermogenic gene expression and beige adipogenesis. Although only one study (Artsi et al.) explicitly aimed to evaluate adipocyte browning (25), the remaining studies reported browning-related features in the context of broader investigations into skeletal regulation of systemic metabolism, G protein signaling, and Wnt pathway modulation.

**Table 1:** Comparative mechanistic overview of the five included studies investigating osteocyte–adipocyte crosstalk and browning regulation.

Pathway/Effect	Model(s)	Depot(s)	Thermogenic evidence	Osteocyte specificity	Sex & age	Ref
Activating Sirt1 ↓ PGC1α▴ Sost▾	Sirt1^Δ/+^ mice (129/Sv), C3H10T1/2 cell line, human BM-MSCs	Tibial & vertebral BMAT (mouse); femoral BM-MSC (human)	▾Pgc1α, ▾Prdm16 in tibial BMAT ▴PGC1α ▴Ucp1 with SRT3025 or anti-sclerostin	MSC-targeted; ^*^not osteocyte-specific	Male & female, 5–7 months Female human patients, 68 ± 9.3 years	(25)
PPARγ deletion (Dmp1-Cre)	Dmp1-Cre; PPARγ^-/-^ mice, 3T3L1 cell line	BAT, iWAT	▴Ucp1 for iWAT and BAT ▴Prdm16 for iWAT ▴O_2_ consumption for PPARγ-/-	Oste lineage-specific (^*^not osteocyte-specific)	Male, 3–6 months, Mice	(34)
Gsα deletion ↓ Sost▴ ↓ WAT browning	DMP1-Cre^(+)^:Gsα^(flox/flox)^, OC-Cre^(+)^:Gsα^(flox/flox)^, and DMP1-ERT2-GsαKO; Ocy454 cell line	iWAT& gWAT, BAT	▴Ucp1 ▴Pgc1α conditioned media from Ocy454 with Gsα loss promoted beige adipogenesis	Osteolineage-specific *in vivo*; osteocyte-specific *in vitro*	Male and female, 8–18 weeks (depending on model used), Mice	(23)
Sclerostin Wnt inhibition	Sost^–/–^ mice, anti-Sost Ab, adipocyte cultures + sclerostin	Inguinal, visceral WAT; *in vitro* adipocytes	▴Ucp1 ▴Pgc1α Wnt inhibition promotes beige features	Systemic KO/Ab; not osteocyte-specific	Male, 8–10 weeks, Mice	(35)
Sclerostin + Exercise	C57bl6 mice + treadmill running ± recombinant sclerostin	Epididymal WAT	No effect on Ucp1 or PGC1α for iWAT ▾adipocyte size with exercise only	No cell-specific targeting	10 weeks old male, Mice	(36)

Sirt1^Δ/+^:Heterozygous deletion of the Sirtuin 1 gene, C3H10T1/2: Mouse mesenchymal stem cell line, SRT3025: Small molecule activator of Sirt1, Dmp1-Cre: Dentin matrix protein 1 promoter-driven Cre recombinase, 3T3L1: Mouse preadipocyte cell line, DMP1-ERT2-GsαKO: Inducible osteocyte-specific Gsα knockout model, Ocy454: Osteocyte-like cell line, Sost^–/–^ mice: Sclerostin knockout mice, Anti-Sost Ab: Antibody against sclerostin, C57BL/6J mice: are resistant to audiogenic seizures, have a relatively low bone density.▾: Decrease, ▴Increase. *Not osteocyte-specific: Osteolineage-specific models may confound osteoblast-derived signals with osteocyte function.

Brun et al. investigated the effects of PPARγ deletion in mature osteoblasts and osteocytes, finding increased Ucp1, Peroxisome proliferator-activated receptor gamma coactivator 1-alpha (Pgc1α), and PR domain containing 16 (Prdm16) expression in both inguinal white adipose tissue (iWAT) and brown adipose tissue (BAT), along with enhanced energy expenditure (34). Fulzele et al. explored that constitutive or inducible loss of Gsα in osteolineage cells resulted in increased circulating irisin and upregulation of thermogenic markers in subcutaneous and visceral WAT (23). Kim et al. examined the role of sclerostin using both a global constitutive Sost knockout (Sost^−^/^−^) mouse model and a post-natal systemic overexpression model in which sclerostin was elevated via liver-targeted AAV-Sost delivery. In this context, they demonstrated that sclerostin influences adipocyte metabolism and promoted expression of thermogenic and browning-associated genes in cultured preadipocytes and adipose tissues. *In vivo*, Sost deficiency reduced fat mass and increased thermogenic markers, whereas overexpression caused adipocyte hypertrophy and fat accumulation (35). Kurgan et al. assessed the effect of recombinant sclerostin under exercise conditions, reporting morphological but not molecular changes in adipose tissue (36). Artsi et al. focused on Sirt1 activation and sclerostin suppression in mouse and human bone marrow-derived cells, finding consistent increases in UCP1, PGC1α, and PRDM16 (25).

Fat depots analyzed included subcutaneous and visceral WAT, bone marrow adipose tissue (BMAT), and BAT. Some studies demonstrated strong evidence of thermogenesis via gene and protein expression, histology, and functional outcomes, while others relied primarily on mRNA markers or morphological assessments. Although most studies used osteolineage-specific models (e.g., Dmp1-Cre), none selectively targeted osteocytes, leaving open the possibility of confounding effects from mature osteoblasts or other mesenchymal cells. Only Artsi et al. included human samples, highlighting the translational potential of this emerging field (25).

A comparative overview of the included studies is provided in **Table 1**. This summary outlines the major osteocyte-related pathways investigated, experimental models, adipose depots examined, thermogenic readouts, and key limitations related to cell specificity and study design.

### 3.1 Methodological rigor and risk of bias

All five included studies demonstrated low risk in baseline comparability and outcome completeness (**Table 2**). However, most domains, including sequence generation, allocation concealment, random housing, and blinding, were not explicitly reported, resulting in “unclear” risk ratings. Only Kurgan et al. described structured allocation and blinding (36), whereas Artsi et al. acknowledged confounding due to tissue heterogeneity (25), resulting in a high-risk judgment for “other sources of bias”. Notably, none of the included studies employed osteocyte-specific inducible models *in vivo*, which limits the ability to attribute observed effects exclusively to osteocytes rather than to broader osteolineage activity.

**Table 2:** SYRCLE risk of bias assessment for the five included animal studies. Each cell represents one of ten bias domains as judged per study.

Bias domain	Artsi et al.	Brun et al.	Fulzele et al.	Kim et al.	Kurgan et al.
1. Sequence generation (randomization)	Unclear	Unclear	Unclear	Unclear	Low
2. Baseline characteristics	Low	Low	Low	Low	Low
3. Allocation concealment	Unclear	Unclear	Unclear	Unclear	Unclear
4. Random housing	Unclear	Unclear	Unclear	Unclear	Unclear
5. Blinding of caregivers/investigators	Unclear	Unclear	Unclear	Unclear	Low
6. Random outcome assessment	Unclear	Unclear	Unclear	Unclear	Unclear
7. Blinding of outcome assessors	Unclear	Unclear	Unclear	Unclear	Low
8. Incomplete outcome data	Low	Low	Low	Low	Low
9. Selective outcome reporting	Unclear	Unclear	Unclear	Unclear	Unclear
10. Other sources of bias	Hight	Unclear	Unclear	Unclear	Unclear

**Green** = Low risk, **Grey** = Unclear risk, **Red** = High risk.

While the high frequency of ‘unclear’ ratings suggests a need for improved reporting standards in the field, the consistency of browning outcomes across heterogeneous models—ranging from genetic mouse knockouts to human-derived cell cultures, suggests that the core biological findings are robust and independent of specific reporting gaps.

## 4 Discussion

Osteocytes have emerged as endocrine regulators whose signals influence tissues well beyond bone (3, 9, 37, 38). Among these signals, sclerostin, Sirt1, PPARγ, and BMP7 have been implicated as modulators of adipocyte phenotype and energy metabolism (27–29, 34, 39–41). Across the five preclinical studies included in this review, these pathways converge on a shared outcome: modulation of thermogenic remodeling in adipose tissue, either through induction of beige adipocytes or through repression of oxidative and mitochondrial programs (23, 25, 34–36).

A key emerging insight is that these osteocyte-derived signals operate within a redox-sensitive framework. Both osteocytes and adipocytes are metabolically active cells whose function is tightly regulated by mitochondrial activity, NAD^+^ availability, and reactive oxygen species (ROS) (20, 21, 42). As a result, endocrine communication between bone and fat is not merely hormonal but deeply intertwined with oxidative stress, antioxidant defenses, and redox-mediated transcriptional control.

This context is essential, because the included studies demonstrated divergent thermogenic outcomes depending on the signaling environment. Some models showed sclerostin- or PPARγ-driven promotion of beige adipogenesis (23, 34), whereas others reported suppression of thermogenic gene expression when sclerostin was elevated or overexpressed (35). These discrepancies suggest that osteocyte–adipocyte signaling is not unidirectional but instead shaped by genetic background, depot specificity, and critically, by the local redox milieu, which influences both osteocyte secretory output and adipocyte responsiveness.

### 4.1 Sclerostin: a context-dependent regulator of adipose thermogenesis

**Figure 3:**
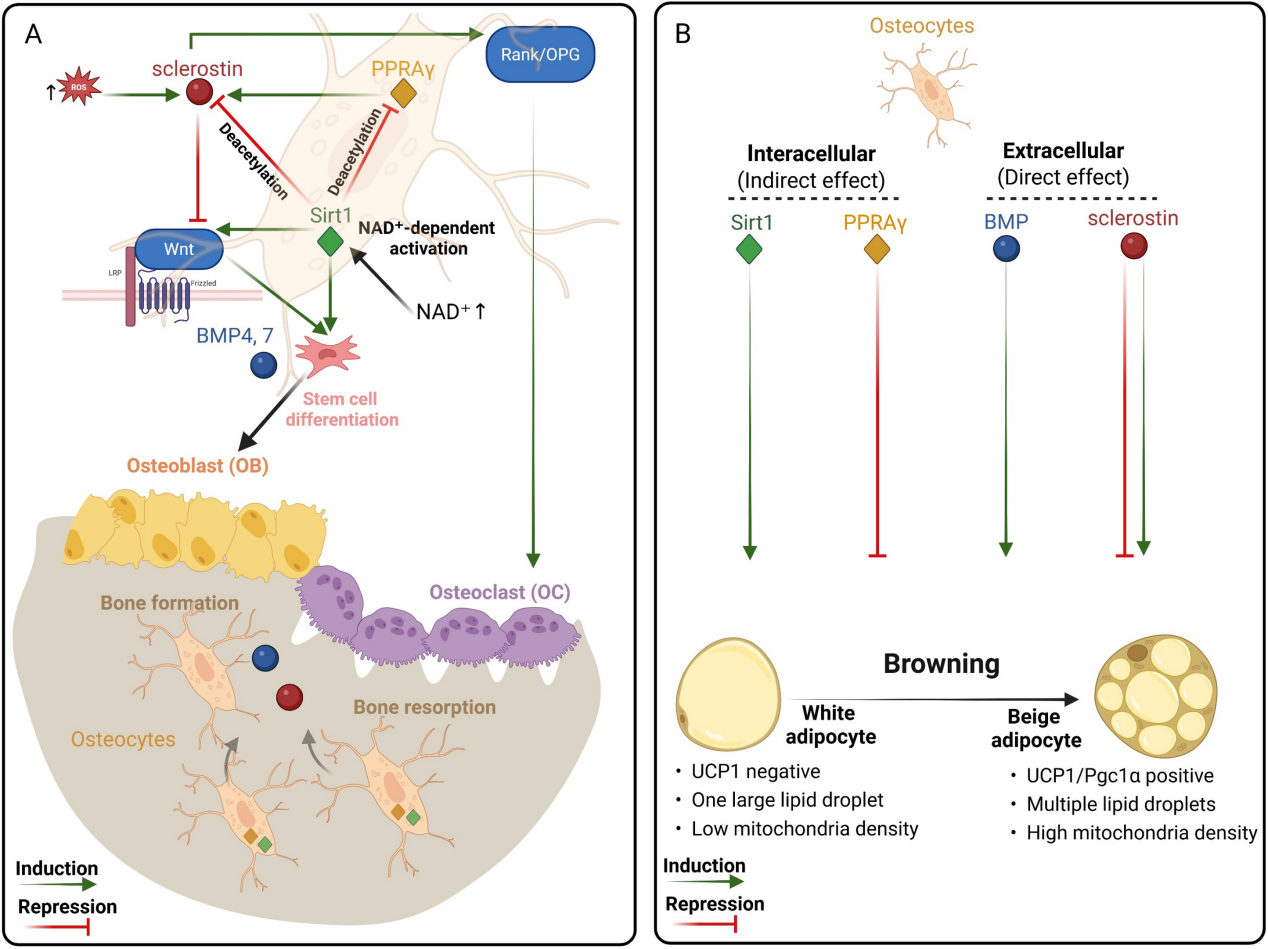
Osteocyte-derived regulatory network linking bone metabolism, redox balance, and adipocyte browning. **(A)** Crosstalk among Sclerostin, PPARγ, SIRT1, and BMPs coordinates bone remodeling. In osteocytes, reactive oxygen species (ROS) activate Sost expression, enhancing sclerostin release and bone resorption, whereas Sirt1-driven antioxidant signaling (via NAD^+^-dependent deacetylation) represses sclerostin, sustaining osteoblast differentiation and bone formation. BMP7 further supports osteogenic and thermogenic programs, integrating Wnt signaling. **(B)** Dual effects of osteocyte-derived mediators (Intracellular vs Extracellular) on adipocyte phenotypes. White adipocytes are prone to ROS accumulation and metabolic stress, whereas beige adipocytes display higher mitochondrial density and enhance antioxidant defense. The presence of UCP1 in beige adipocytes facilitates mitochondrial uncoupling. Created with BioRender.com.

Sclerostin, a Wnt/β-catenin inhibitor produced predominantly by osteocytes, emerged across the included studies as a key regulator of bone–fat communication (23, 35, 36). However, its effects on adipocyte thermogenesis differed markedly between models, indicating strong context dependency (**Figure 3A**).

Fulzele et al. investigated the role of sclerostin in adipose thermogenesis using three mouse models with elevated sclerostin levels, generated by constitutive or inducible deletion of Gsα in mature osteoblasts and/or osteocytes. These mice exhibited progressive loss of gonadal WAT (gWAT) and iWAT, with increased multilocular adipocytes in both depots and reduced canonical β-catenin signaling. Thermogenic genes were upregulated in iWAT, and BAT also showed increased Ucp1 expression. *In vitro*, conditioned media from Gsα-deficient osteocytic murine osteocyte-like Ocy454 cells, which secrete high levels of sclerostin, significantly increased UCP1 expression in primary murine adipocytes isolated from subcutaneous WAT. This effect was partially reduced through sclerostin immunodepletion using an antibody. *In vivo*, sclerostin-neutralizing antibody treatment partially reduced UCP1 expression in WAT of Gsα-deficient mice, whereas recombinant sclerostin administration to wild-type mice significantly increased Ucp1 expression in WAT. These findings imply that high circulating sclerostin levels in a Gsα-deficient can promote beige adipogenesis. However, the experiments were conducted in pathophysiological models with markedly increased circulating sclerostin, and Cre drivers targeting both osteoblasts and osteocytes, which may limit extrapolation to physiological settings and osteocyte-specific effects (23).

In contrast, Kim et al. examined sclerostin function under more physiological conditions using Sost knockout mice, transgenic Sost overexpressors, and anti-sclerostin antibody treatment. They reported that deletion or pharmacological inhibition of sclerostin reduced body weight and total fat mass, increased whole-body oxygen consumption (VO_2_), and upregulated thermogenic and oxidative genes predominantly in subcutaneous (inguinal) WAT (35). Conversely, sclerostin overexpression increased adiposity and impaired glucose tolerance. *In vitro*, recombinant sclerostin promoted lipid accumulation and adipogenic gene expression (PPARγ, C/EBPα) in 3T3-L1 preadipocytes while reducing oxidative metabolism in mature adipocytes (43). These results position sclerostin as a negative regulator of browning, in contrast to the pro-thermogenic phenotype observed in the severe Gsα-deficiency model described by Fulzele et al. It should be noted, however, that in this model changes in SOST expression occur as part of a broader spectrum of molecular alterations induced by Gsα deficiency, and additional factors affected by Gsα loss are also likely to contribute to the observed adipose phenotype. This apparent divergence underscores that sclerostin’s impact on adipose phenotype is highly context-dependent and may be shaped by differences in genetic background, the magnitude and duration of sclerostin elevation, depot specificity, and systemic metabolic.

Furthermore, Kurgan et al. examined the interaction between sclerostin and exercise-induced adaptations. In treadmill-trained male mice, sclerostin administration attenuated the exercise-induced reduction in adipocyte size in subcutaneous WAT but did not alter UCP1 or PGC1α expression levels (36). Exercise itself decreased circulating sclerostin and reduced oxidative stress, indicating that the metabolic benefits of training may involve simultaneous suppression of sclerostin and restoration of redox balance. Thus, under normal physiological loading, sclerostin primarily affects adipocyte morphology more readily than browning-related gene expression, suggesting a threshold-dependent effect. These findings indicate that moderate, physiologically relevant changes in sclerostin may be insufficient to drive thermogenic reprogramming.

Sost expression is also regulated by cellular redox status. Studies in osteocytes and osteocyte-like cells show that increased ROS levels enhance Sost expression through ROS-dependent suppression of Sirt1 (44). Changes in oxygen tension and oxidative metabolism further modulate Sost through epigenetic regulation in osteocytes, linking hypoxia and redox imbalance to altered sclerostin output (45). Antioxidant pathways, particularly NRF2 activation, oppose these effects and maintain osteocytic redox homeostasis, indirectly supporting low-Sost states (46). Thus, oxidative stress biases toward high sclerostin and Wnt inhibition, whereas a reduced or antioxidant-dominant environment suppresses Sost. This redox-sensitive toggle provides a plausible explanation for why sclerostin promotes browning in some models yet suppresses it in others: osteocyte redox status conditions the endocrine output of Sost, which in turn shapes adipocyte phenotype.

Artsi et al. further support this framework by showing that pharmacologic activation of Sirt1 in murine and human BM-MSCs represses Sost expression through histone deacetylation and concurrently enhances UCP1, PGC1α, and PRDM16 expression (25). Anti-sclerostin antibody reproduced these effects, establishing sclerostin as a negative regulator of marrow adipocyte browning (25). While these data point to a Sirt1→Sost axis, broader Sirt1-dependent antioxidant signaling belongs conceptually to the next subsection (4.2) and is not elaborated here. Importantly, the inclusion of evidence from human-derived bone marrow stromal cells (Artsi et al.) provides a critical translational link, suggesting that the osteocyte-driven browning mechanisms observed in rodent models may be conserved in human physiology, thereby increasing the reliability of the overall conclusion.

Additional mechanistic support comes from Ukita et al., who showed in 3T3-L1 preadipocytes that recombinant sclerostin enhances adipogenic differentiation via upregulation of Pparγ, C/EBPβ, and Adiponectin, and suppression of Wnt family member 3A (Wnt3a) and TAZ (43). Though excluded from our systematic review due to the absence of thermogenic outcomes, this study highlights the adipogenic potential of sclerostin *in vitro* (43).

Taken Together, these findings highlight the complex and model-dependent nature of sclerostin’s role in adipocyte thermogenesis. Factors such as the specific adipose depot, systemic signals (e.g., irisin, Gsα), and upstream osteocyte signaling context may modulate whether sclerostin promotes or suppresses browning. This duality underscores the need for studies employing osteocyte-specific tools and longitudinal analyses to unravel local and systemic effects of sclerostin on energy metabolism.

### 4.2 Nuclear and epigenetic modulators of osteocyte-adipocyte communication

Sirt1 and PPARγ represent two central, redox-sensitive transcriptional regulators that integrate osteocyte function with adipocyte phenotype. Sirt1 promotes osteoblast differentiation, represses the osteocyte-derived Wnt inhibitor sclerostin via histone deacetylation, and restricts marrow adiposity (**Figure 3A**), while PPARγ is essential for adipogenesis and energy storage (40, 47, 48). Beyond their roles in adipogenesis and bone remodeling, osteocytic PPARγ and PPARα have been shown to influence cell bioenergetics, bone integrity, and systemic energy metabolism, underscoring the broader metabolic functions of osteocyte nuclear receptors (26, 49). Importantly, Sirt1 can directly deacetylate and repress PPARγ activity, shifting adipocytes toward a beige phenotype (**Figure 3A**) (24). In the skeletal niche, these pathways converge in osteolineage cells and osteocytes to influence both bone remodeling and adipocyte thermogenic programming, positioning them as central modulators of osteocyte–adipocyte communication.

Sirt1 functions as an NAD^+^-dependent metabolic sensor whose activity is directly regulated by oxidative stress. Caito et al. demonstrated that Sirt1 undergoes post-translational inhibition under oxidative and carbonyl stress, reducing its deacetylase activity (50). Conversely, in settings where Sirt1 remains active, it deacetylates Forkhead Box O3a (FOXO3a) and PGC1α—forming a FOXO3a:PGC1α transcriptional complex that induces antioxidant enzymes such as MnSOD, catalase, Peroxiredoxins, and thioredoxin/thioredoxin-reductase-2 (51). This antioxidant program reduces ROS accumulation and supports mitochondrial resilience, establishing Sirt1 as a key redox-protective regulator within osteolineage cells.

In the study by Brun et al., conditional deletion of PPARγ in mature osteoblasts and osteocytes (Dmp1-Cre) uncovered a clear age-dependent phenotype: while young adult mice (3 months) showed no overt metabolic differences, older knockouts (6 months) exhibited reduced fat mass, increased whole-body VO_2_, and improved glucose tolerance. Positron emission tomography–computed tomography imaging (PET-CT) revealed elevated glucose uptake in BAT, consistent with enhanced thermogenic activity. At the tissue level, iWAT contained multilocular adipocytes with higher UCP1 expression, in contrast to the larger unilocular lipid droplets seen in controls. BAT in KO mice also displayed smaller lipid droplets and an amplified thermogenic gene program. Beyond adipose depots, hepatic lipid accumulation was diminished, pointing to broader systemic metabolic benefits. Mechanistically, conditioned media from osteoblast–osteocyte cultures of KO mice induced UCP1 expression in 3T3-L1 preadipocytes, suggesting the involvement of bone-derived secreted factors. Reduced Sost expression further implicated osteocyte-derived sclerostin as part of the signaling axis linking skeletal PPARγ to adipose phenotype. The authors acknowledged a potential limitation as they mentioned that Dmp1-Cre activity can extend beyond osteocytes into skeletal muscle, which may contribute to the systemic metabolic effects observed (34).

Artsi et al. as discussed earlier, expanded on this topic by examining how Sirt1 influences adipocyte thermogenesis in the bone marrow environment. Using Sirt1^Δ/+^ haploinsufficient mice, they observed a significant reduction in thermogenic gene expression specifically in tibial BMAT of female mice, but not in vertebral BMAT or in male mice. These findings highlight the region- and sex-specific effects of Sirt1 on marrow adipose phenotype. In line with a cell-autonomous mechanism, PRDM16 and PGC1α were also downregulated in BM-MSCs derived from Sirt1^Δ/+^ mice during adipogenic differentiation. *In vitro*, pharmacologic activation of Sirt1 with SRT3025 enhanced thermogenic gene expression in murine mesenchymal C3H10T1/2 cells and in human BM-MSCs. This upregulation was accompanied by epigenetic repression of Sost through histone deacetylation. Treatment with a monoclonal anti-sclerostin antibody verified the thermogenic effects, reinforcing a mechanistic link between Sirt1 activity, sclerostin inhibition, and beige adipogenesis. However, the reliance on BM-MSCs, rather than committed or mature adipocytes, and the use of heterogeneous stromal cell populations limit the physiological specificity of these findings (25).

Although not included in our review, Baroi et al. further supports this axis by showing that osteocyte-specific deletion of PPARγ in mice resulted in reduced Sost expression, increased bone mass, and lower marrow adiposity. While browning markers were not assessed in this model, the results reinforce the role of PPARγ as a transcriptional factor of sclerostin in osteocytes (52). Additional indirect evidence comes from Wang et al., who used siRNA to knock down PPARγ in rabbit BMSCs treated with dexamethasone. The intervention blocked adipocyte differentiation and restored osteogenic gene expression, emphasizing the centrality of PPARγ in mesenchymal lineage allocation. Though this study did not explore thermogenesis or osteocyte-specific mechanisms, it illustrates the broader regulatory role of PPARγ in the bone–fat axis (53).

Collectively, these findings identify Sirt1 and PPARγ as redox-sensitive transcriptional nodes that coordinate bone–fat communication. Sirt1 acts as an antioxidant and mitochondrial stabilizer that suppresses sclerostin, whereas PPARγ amplifies oxidative and adipogenic signaling when dysregulated. Their opposing roles define a redox-controlled rheostat governing whether osteocytes promote bone formation and thermogenic remodeling or favor adipogenesis and metabolic dysfunction. Future studies that target these regulators specifically in osteocytes, while accounting for sex, depot, and context-specific responses, will be essential for refining therapeutic strategies.

### 4.3 BMP signaling as a modulatory pathway in adipose browning

BMPs are pivotal regulators of adipocyte development, with BMP2, BMP4, BMP7, and BMP8 playing distinct yet complementary roles in the browning and thermogenic activation of adipose tissue. BMP7 is established as a master inducer of brown adipogenesis, driving the commitment of mesenchymal progenitors toward a brown fat fate by activating PRDM16 and UCP1 (54). While BMP4 was historically associated with white adipocyte commitment, recent evidence demonstrates that it also facilitates the white-to-brown transition in human adipose stem cells and contributes to the recruitment of thermogenic beige adipocytes (55). Furthermore, BMP8 has emerged as a key regulator of energy balance, acting both centrally in the brain to increase sympathetic output and peripherally to enhance the thermogenic response of brown and beige adipocytes to adrenergic stimulation (56, 57). Together, these BMP family members orchestrate a complex signaling network that governs the formation, maturation, and metabolic activity of thermogenic adipose depots.

Kim et al. reported that recombinant sclerostin suppressed oxidative metabolism and thermogenic gene expression in adipocytes, and this suppression was associated with reduced BMP4 expression, suggesting that sclerostin may inhibit browning partly by downregulating BMP4 (35). Conversely, Sost deletion or antibody-mediated Sost inhibition increased oxygen consumption and thermogenic markers, implying BMP4 may contribute to the thermogenic shift observed when sclerostin is suppressed (35).

Brun et al. speculated that enhanced thermogenesis in PPARγ-deficient osteoblast/osteocyte-lineage mice could involve increased BMP7 signaling, although BMP7 itself was not measured (34). This remains hypothetical but aligns with established roles for BMP7 in promoting UCP1, PGC1α, and mitochondrial biogenesis in white adipocytes.

BMP7 serves as a key regulator of mitochondrial function and oxidative balance. BMP7 signaling promotes mitochondrial biogenesis, activates oxidative phosphorylation, and induces antioxidant enzymes such as catalase and glutathione peroxidase, thereby reducing ROS generation during high-energy flux (58). These effects sustain thermogenic capacity and protect adipocytes from oxidative overload.

Beyond the included studies, several independent investigations support a role for BMP7 in thermogenesis. Boon et al. demonstrated that BMP7 treatment in mice increased UCP1 expression, lipid oxidation, and mitochondrial content in WAT under subthermoneutral conditions. These effects could not be seen under thermoneutral conditions (29). Casana et al. applied Adeno-associated virus-mediated (AAV-mediated) BMP7 gene therapy to obese mice and reported improved insulin sensitivity, reduced fat mass, and enhanced browning marker expression (41). These metabolic improvements were accompanied by reduced oxidative stress markers and increased mitochondrial antioxidant capacity, linking BMP7-induced thermogenesis to systemic redox homeostasis. While neither study involved osteocyte-specific manipulation, both highlight the thermogenic potential of BMP7 and support its inclusion in the mechanistic framework linking bone-derived signals to adipose phenotype regulation.

Whether osteocytes physiologically secrete BMP7 in quantities sufficient to affect adipose tissue remains unresolved. However, existing data suggest that BMP7 expressions in bone and mesenchymal cells is redox-responsive, increasing under controlled oxidative signaling but decreasing under chronic oxidative stress or inflammation (58). Taken together, these findings position BMP7 as a redox-responsive mediator capable of linking osteocyte signaling to adipocyte mitochondrial remodeling. While evidence connecting osteocytes and BMP7-driven browning is currently indirect, BMP7’s established role in both oxidative regulation and thermogenic activation supports its inclusion as a candidate effector in the bone–fat endocrine axis.

### 4.4 Broader skeletal–adipose crosstalk beyond canonical osteocyte-mediated browning

Beyond classical regulators such as sclerostin and BMPs, emerging evidence indicates that osteocytes secrete additional bioactive molecules capable of modulating adipose tissue phenotype and thermogenic potential. Among these, Receptor Activator of Nuclear Factor-κB Ligand (RANKL) and prostaglandin E2 (PGE_2_) have gained attention as potential mediators of osteocyte–adipocyte crosstalk. Although these factors were originally characterized by their roles in bone remodeling and inflammatory signaling, accumulating data suggests that they may also influence adipocyte differentiation and metabolic programming. The current evidence supporting the involvement of RANKL and PGE_2_ in adipose tissue browning.

RANKL is best known for its essential role in osteoclastogenesis; however, recent studies suggest that RANKL signaling may also influence adipose tissue biology. Experimental evidence indicates that activation of the RANK/RANKL pathway can promote the differentiation of preadipocytes toward a beige adipocyte phenotype. *In vitro* stimulation of preadipocytes with RANKL has been shown to enhance the expression of thermogenic markers, including UCP1, and to induce a multilocular lipid droplet morphology characteristic of beige adipocytes. These cellular changes are accompanied by increased mitochondrial activity and elevated respiratory capacity, suggesting a functional shift toward a more oxidative and energy-expending phenotype. Importantly, these effects appear to be most pronounced in adipocyte precursor cells rather than in fully differentiated white adipocytes, indicating that RANKL primarily acts during the early stages of beige adipocyte commitment and differentiation. *In vivo*, RANKL administration was reported to increase whole-body energy expenditure and to confer partial resistance to high-fat diet–induced weight gain, further supporting a potential metabolic role for this pathway (59).

PGE_2_, a lipid mediator produced through cyclooxygenase activity, has also emerged as a regulator of adipose tissue plasticity. Recent work has identified the PGE_2_–E-prostanoid receptor 3 (EP3) axis as an important modulator of brown and beige adipocyte development. Activation of EP3 signaling during adipocyte differentiation has been shown to promote the expression of thermogenic genes and to support the acquisition of a brown-like phenotype. Mechanistically, PGE_2_/EP3 activation enhances the stability of Zfp410 mRNA via WTAP-mediated m6A RNA methylation, thereby facilitating transcriptional programs required for brown adipogenesis. Disruption of this signaling axis impairs the browning process, leading to reduced thermogenic capacity. In animal models, inhibition or genetic deficiency of EP3 signaling has been associated with defective brown adipose tissue formation, increased susceptibility to high-fat diet–induced obesity, and worsened insulin resistance. These findings highlight PGE_2_ as a potential paracrine factor capable of influencing adipocyte metabolic fate and systemic energy homeostasis (60).

Collectively, these studies suggest that RANKL and PGE_2_ represent additional layers of osteocyte-derived regulation that may contribute to adipose tissue browning and metabolic adaptation. While the current evidence remains limited and is largely derived from experimental models, both mediators appear capable of modulating early adipogenic differentiation and thermogenic gene expression through distinct molecular mechanisms. Further investigation is required to determine whether osteocyte-derived RANKL and PGE_2_ exert physiologically relevant endocrine effects on adipose depots in humans, and how these pathways interact with established regulators such as sclerostin, Wnt, and BMP signaling.

In addition to the studies included in this review, several reports have highlighted broader aspects of skeletal cell–adipose tissue crosstalk that are relevant to, but distinct from, osteocyte-mediated browning. For example, Li et al. demonstrated that bone could regulate systemic white adipose tissue browning through Schnurri-3–dependent secretion of SLIT2, primarily via osteoblast-lineage cells, supporting the concept that skeletal tissues can exert endocrine control over adipose thermogenic programming (61). Furthermore, Fairfield et al. reported that the osteocyte-derived molecule sclerostin promotes bone marrow adipogenesis, emphasizing an important role of osteocytes in regulating adipocyte formation within the marrow niche (61). However, bone marrow adipogenesis represents a biological process that is mechanistically different from the browning of classical white adipocytes, which was the specific focus of the present review. Consistent with this distinction, current consensus guidelines on bone marrow adiposity highlight that bone marrow adipocytes constitute a unique adipocyte subtype with specialized functions (62). Together, these studies provide important context for understanding the diverse interactions between skeletal cells and adipose tissues, but fall outside the scope of our systematic analysis, which was restricted to evidence directly addressing osteocyte-driven regulation of white adipocyte browning.

### 4.5 Clinical implications and translational signals

Translating preclinical insights on osteocyte–adipocyte crosstalk into human physiology has become increasingly relevant as bone- and fat-targeted therapies enter widespread clinical use. Sclerostin inhibitors, such as romosozumab, are approved for the treatment of postmenopausal osteoporosis and provide a unique opportunity to assess the systemic consequences of modulating osteocyte-derived signals (7, 63). Data from the FRAME bone biopsy substudy showed that romosozumab did not alter iliac crest bone marrow adiposity over 12 months, despite marked anabolic effects on bone mass and structure (64). In contrast, a spectroscopy study in estrogen-deficient rabbits found that sclerostin antibody prevented marrow fat accumulation and improved bone architecture (65), highlighting potential species- and model-dependent differences. These observations suggest that while anti-sclerostin therapy is effective at stimulating bone formation, its impact on BMAT and systemic metabolism in humans remain uncertain.

Sost-expression itself appears highly sensitive to oxidative stress. Elevated ROS in osteocytes can upregulate Sost, enhancing bone resorption, whereas antioxidant pathways driven by Sirt1 or mechanical loading suppress sclerostin release (40, 44, 50, 66). Thus, therapies that reduce oxidative stress, either pharmacologically or through lifestyle interventions, may indirectly restore the osteocyte redox balance and reinforce bone formation.

Additional evidence underscores the complexity of this axis in clinical populations. In postmenopausal women, circulating sclerostin correlated with lean and fat mass but not directly with bone marrow adiposity assessed by MRI (67). In young women, circulating sclerostin and SIRT1 were differentially associated with bone quality depending on adiposity status (68), while in adults with prediabetes, sclerostin and irisin showed sex-specific interactions in relation to adiposity (69). These findings imply that oxidative stress and NAD^+^ metabolism, which differ between sexes and metabolic states, may modify the responsiveness of bone-fat signaling pathways.

Moreover, exercise is a physiological modulator of both redox status and osteocyte signaling (70). Short-term sprint-interval training in obese men reduced sclerostin protein content in subcutaneous adipose tissue, increased Wnt signaling, and improved oxidative metabolism (71). Exercise-induced shear stress in bone transiently elevates ROS, activating adaptive antioxidant responses (NRF2, Sirt1) that favor osteogenic and thermogenic signaling. This suggests that the metabolic benefits of exercise extend beyond the skeletal system to systemic redox remodeling mediated by osteocyte-derived factors.

Collectively, these clinical and translational observations emphasize that osteocyte-derived mediators such as sclerostin and SIRT1 intersect with pathways targeted in both osteoporosis and metabolic disease. While current data indicate that skeletal and metabolic responses to osteocyte-directed therapies may not always align, systematic assessment of marrow fat, circulating osteocyte-derived proteins, and depot-specific adipose changes in treated patients is now essential. Such approaches will help determine whether pharmacologic interventions designed for bone or metabolic health inadvertently affect the other tissue—and may ultimately reveal opportunities for dual-targeted therapies.

**Figure 4:**
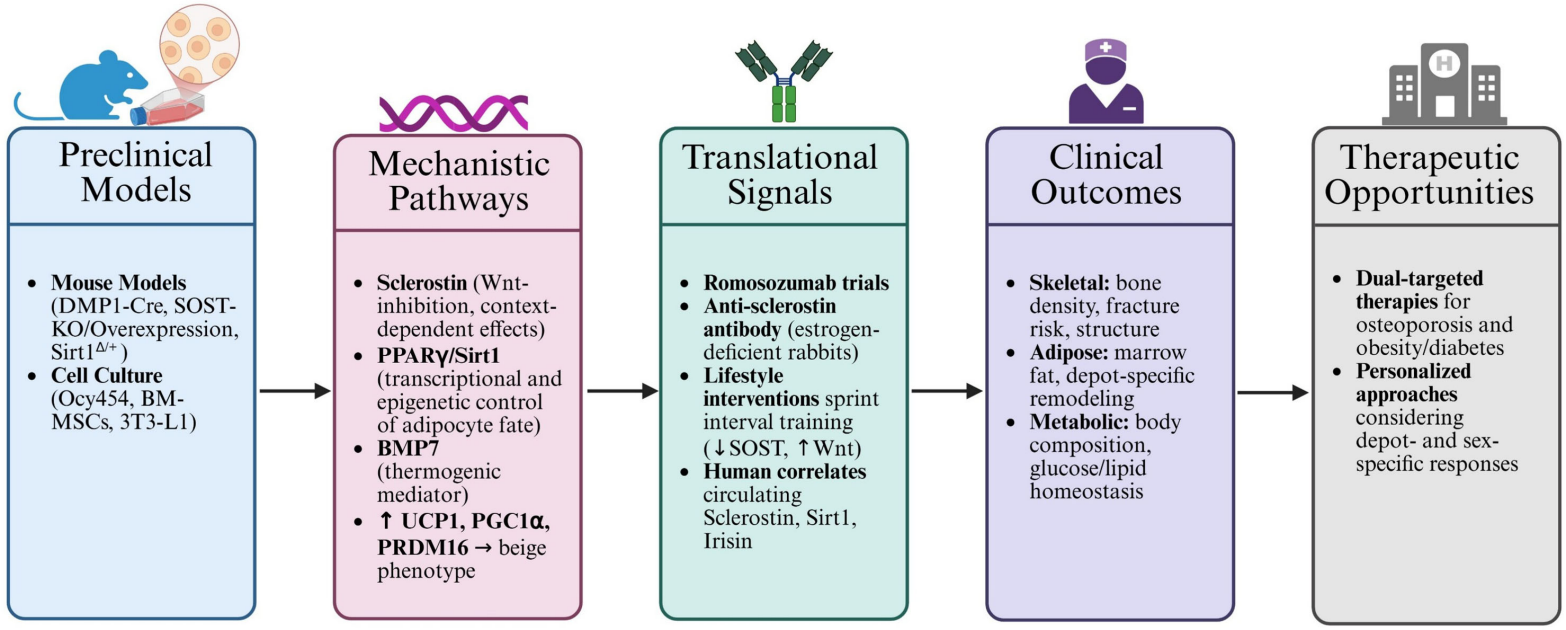
Bench-to-bedside translational pathway linking osteocyte signals to clinical outcomes, illustrating the translational continuum of osteocyte-adipocyte research from mechanistic discovery to potential therapeutic application. These data position osteocyte-adipocyte crosstalk as a promising target for dual-targeted therapies addressing both skeletal fragility and metabolic disease. Created with BioRender.com.

**Figure 4** illustrates the translational continuum from mechanistic discoveries in osteocyte-adipocyte signaling to potential therapeutic applications addressing both skeletal fragility and metabolic dysfunction.

### 4.6 Strengths and limitations of the evidence base

The available studies provide important biological signals suggesting that osteocytes may influence adipose thermogenesis, but these findings are better viewed as hypothesis-generating than confirmatory. All five studies included in this review incorporated *in vivo* experiments, often supported by *in vitro* assays, and several converged on shared thermogenic pathways, including UCP1, PGC1α, and PRDM16 induction. Nevertheless, incomplete reporting of randomization, blinding, and housing conditions introduces uncertainty about reproducibility. In applying the SYRCLE tool, domains were rated “unclear” whenever these procedures were not explicitly described, reflecting reporting insufficiency rather than evidence of high-risk conduct; thus, these ratings indicate reduced transparency (and residual uncertainty in internal validity) rather than demonstrated bias, and the synthesis is framed as hypothesis-generating rather than confirmatory (**Table 2**). A major conceptual limitation is that most genetic models targeted osteolineage broadly rather than osteocytes specifically, reducing the ability to attribute observed phenotypes to osteocyte-derived signals alone. Most experiments were also relatively short in duration, leaving unclear whether depot-specific adipose remodeling translates into sustained systemic metabolic effects.

## 5 Concluding remarks, limitations, and future perspectives

This Systematic Review advances the hypothesis that osteocytes act as endocrine regulators of adipose thermogenesis. Evidence from five preclinical studies indicates, but does not yet establish, that osteocyte-derived signals, including sclerostin, PPARγ, Sirt1, and BMP7, may influence the plasticity of white adipose tissue. These factors appear to modulate thermogenic remodeling through diverse mechanisms, ranging from Wnt pathway inhibition to transcriptional control and systemic mediators such as irisin. Collectively, these findings suggest a possible role for osteocytes in systemic energy metabolism, although this claim remains debated due to methodological limitations, context-dependency, and translational uncertainties.

Importantly, study outcomes were not consistent but varied across models. The effects of osteocyte-derived signals differed depending on adipose depot, animal age, sex, and the specific genetic or pharmacologic strategies used. Such variability underscores the importance of considering depot-specific metabolic microenvironments and sex-dimorphic endocrine signaling in preclinical design. Without this granularity, findings risk overgeneralization and may obscure clinically relevant mechanisms. For example, BMAT appears to respond differently than subcutaneous or visceral depots, and female animals often show distinct metabolic responses compared with males.

Future research should prioritize osteocyte-specific models, harmonized definitions of browning readouts, and longitudinal systemic analyses. Defining the endocrine contributions of osteocytes to energy balance remains an open question but could reshape our understanding of bone-fat crosstalk. Early translational signals from anti-sclerostin therapy, marrow adiposity studies, and exercise interventions suggest potential clinical relevance, yet effects on adipose phenotype are incompletely defined. Addressing these gaps will determine whether targeting osteocyte-adipocyte signaling can deliver dual benefits for skeletal and metabolic health (see Outstanding Questions). Future research should also explicitly integrate redox biology into osteocyte–adipocyte investigations. This includes quantifying ROS and NAD^+^/NADH ratios, assessing mitochondrial respiration, and employing osteocyte-specific genetic models with redox reporters to dissect causality. Translational studies should evaluate whether therapies targeting oxidative stress, such as Sirt1 activators, NAD^+^ boosters, antioxidants, or controlled exercise regimens, can modulate sclerostin signaling and adipose browning in humans.

## 6 Outstanding questions

- What are the specific physiological or pathological conditions under which osteocytes promote or inhibit adipocyte browning?
- How do sex, age, and adipose depot location affect the response to osteocyte-derived signals?
- Do osteocytes actively secrete browning mediators such as BMP7 *in vivo*, and under what stimuli?
- What is the relative contribution of osteocytes compared to other bone cells in regulating systemic energy metabolism?
- Can osteocyte-specific genetic models help disentangle endocrine from local paracrine effects on adipose tissue?
- Could targeting osteocyte-adipocyte crosstalk lead to dual therapeutic benefits for metabolic and skeletal disorders?
- Under which physiological or pathological conditions (e.g., aging, hypoxia, obesity) do osteocytes promote or suppress adipocyte browning?
- Could pharmacologic modulation of redox pathways (e.g., Sirt1 activators, NAD^+^ precursors, mitochondrial antioxidants) enhance osteocyte–adipocyte communication and improve bone–fat homeostasis?

## Data Availability

The original contributions presented in the study are included in the article and its **Supplementary Material**. Further inquiries can be directed to the corresponding authors. As this is a systematic review, all analyzed data are derived from previously published studies, which are cited within the manuscript.

## Glossary

AAV: Adeno-associated virus: A viral vector frequently used for stable gene delivery *in vivo*--BAT, Brown adipose tissue: Thermogenic fat depot rich in mitochondria that dissipates energy as heat via UCP1
BM-MSC: Bone marrow mesenchymal stem cell: Multipotent progenitor cell within bone marrow capable of differentiating into osteoblasts, adipocytes, and chondrocytes
BMP7: Bone morphogenetic protein 7: A member of the TGF-β
CXCL14: C-X-C Motif Chemokine Ligand 14: A batokine involved in immune cell recruitment and energy regulation
FGF21: Fibroblast Growth Factor 21: An endocrine hormone (also a batokine) that enhances thermogenic activity and metabolic health
gWAT: Gonadal WAT: a visceral fat depot located in the gonadal region, commonly studied in rodent models of metabolism
Gsα: G stimulatory protein alpha subunit: A signaling molecule that couples many G−protein–coupled receptors (GPCRs) to adenylyl cyclase activation
IL-6: Interleukin-6: A cytokine with roles in inflammation and metabolism
iWAT: Inguinal WAT: a subcutaneous fat depot located in the inguinal region
KO: Knockout: Genetic model in which a specific gene has been deleted or inactivated
BMAT: Bone marrow adipose tissue: Adipocytes located in the bone marrow cavity with distinct endocrine and metabolic properties compared to peripheral fat
NRG4: Neuregulin-4: A batokine that influences systemic metabolism, particularly liver function
PEG2: Prostaglandin E2: A major lipid mediator (prostanoid) that regulates inflammation, fever, blood flow, and tissue repair
PET-CT: Positron emission tomography–computed tomography: An imaging modality combining metabolic and structural information, often used to quantify glucose uptake
PGC1α: Peroxisome proliferator-activated receptor gamma coactivator 1-alpha: A transcriptional coactivator that regulates mitochondrial biogenesis and thermogenesis
PPARγ: Peroxisome proliferator-activated receptor gamma: A nuclear receptor that governs adipogenesis and energy storage, also expressed in osteocytes
PRDM16: PR domain containing 16: A transcriptional co-regulator critical for the induction of brown and beige adipocyte lineages
RANKL: Receptor Activator of Nuclear Factor Kappa-B Ligand: A protein that plays a critical role in bone metabolism by promoting the differentiation, activation, and survival of osteoclasts
Sirt1: Sirtuin 1: An NAD⁺-dependent deacetylase that regulates metabolism, mitochondrial function, and sclerostin expression
SOST: Sclerostin: An osteocyte-derived Wnt signaling inhibitor that regulates bone formation and influences adipocyte differentiation and thermogenesis
TRP: Transient Receptor Potential: A family of ion channels
UCP1: Uncoupling protein 1: A mitochondrial inner membrane protein that uncouples oxidative phosphorylation to generate heat in brown and beige adipocytes
VO₂: Oxygen consumption: A physiological measure of metabolic rate and energy expenditure
WAT: White adipose tissue: The main fat depot specialized for energy storage, which can undergo browning to acquire thermogenic properties
Wnt: Wingless/Int signaling pathway: A conserved signaling cascade regulating cell fate, bone formation, and adipocyte differentiation
