# Supplementary material for: Osteocytes orchestrate browning: emerging signals in bone-fat crosstalk: a systematic review
Source: Front Endocrinol (Lausanne). 2026 Mar 6;17:1766959. doi: 10.3389/fendo.2026.1766959 (PMC13002408; doi:10.3389/fendo.2026.1766959)
Supplement: Supplementary file 1 [file Supplementaryfile1.docx]

**Osteocytes Orchestrate Browning: Emerging Signals in Bone-Fat Crosstalk**

Houmam Anees^1,2*^, Vahid Jahed^2^, Zahra Sabouri^2^, Reem Jamous^2^, Cristian Pablo Pennisi^3^, Christian Heiss^1,2,4^_,_ and Thaqif El Khassawna^2,5^*

^1^Department of Trauma, Hand and Reconstructive Surgery, Faculty of Medicine, Justus-Liebig-University of Giessen, 35392 Giessen, Germany

^2^Experimental Trauma Surgery, Faculty of Medicine, Justus-Liebig-University of Giessen, 35392 Giessen, Germany

^3^Regenerative Medicine, Department of Health Science and Technology, Faculty of Medicine, Aalborg University, Aalborg, 9260, Denmark.

^4^Biruni University, Istanbul, Türkiye

^5^School of Pharmacy, The University of Jordan, Amman 11942, Jordan

*** Corresponding Authors:** [Houmam.Anees@chiru.med.uni-giessen.de](mailto:Houmam.Anees@chiru.med.uni-giessen.de) (H.Anees); [Thaqif.ElKhassawna@chiru.med.uni-giessen.de](mailto:Thaqif.ElKhassawna@chiru.med.uni-giessen.de) (T. El Khassawna).

**Supplementary Methods**

**Database Search Strategy:**

The following search strategies were used for database retrieval in accordance with PRISMA guidelines.

**PubMed**

(("Osteocytes"[Mesh] OR osteocyte*[tiab] OR sclerostin[tiab] OR Sost[tiab] OR "osteocyte conditioned media"[tiab] OR "osteocyte secretome"[tiab] OR "osteocyte derived extracellular vesicle"[tiab] OR DMP1[tiab] OR Phex[tiab]) AND ("Adipose Tissue, Brown"[Mesh] OR "brown adipose tissue"[tiab] OR "brown fat"[tiab] OR "beige adipocyte*"[tiab] OR browning[tiab] OR thermogenesis[tiab] OR UCP1[tiab] OR "uncoupling protein 1"[tiab] OR PGC1A[tiab] OR "PGC-1α"[tiab] OR PRDM16[tiab] OR CIDEA[tiab] OR DIO2[tiab] OR TMEM26[tiab])) AND ("2000/01/01"[PDAT] : "3000"[PDAT]) AND (english[lang])

**Embase (Ovid):**

('osteocyte'/exp OR osteocyte*:ti,ab,kw OR sclerostin:ti,ab,kw OR 'sclerostin'/exp OR

sost:ti,ab,kw OR 'osteocyte conditioned media':ti,ab,kw OR 'osteocyte secretome':ti,ab,kw OR 'osteocyte derived extracellular vesicle':ti,ab,kw OR dmp1:ti,ab,kw OR phex:ti,ab,kw) AND ('brown adipose tissue'/exp OR 'adipose tissue brown':ti,ab,kw OR 'brown fat':ti,ab,kw OR 'beige adipocyte*':ti,ab,kw OR browning:ti,ab,kw OR thermogenesis:ti,ab,kw OR ucp1:ti,ab,kw OR 'uncoupling protein 1':ti,ab,kw OR pgc1a:ti,ab,kw OR prdm16:ti,ab,kw OR cidea:ti,ab,kw OR dio2:ti,ab,kw OR tmem26:ti,ab,kw) AND [english]/lim AND [2000-2025]/py

**Web of Science Core Collection**

TS=( (osteocyte* OR sclerostin OR Sost OR "osteocyte conditioned media" OR "osteocyte secretome" OR (osteocyte NEAR/3 extracellular NEAR/3 vesicle*) OR DMP1 OR Phex) AND ("brown adipose tissue" OR "brown fat" OR "beige adipocyte*" OR browning OR thermogenesis OR UCP1 OR "uncoupling protein 1" OR PGC1A OR PRDM16 OR CIDEA OR DIO2 OR TMEM26) ) AND LA=(English) AND PY=(2000-2025)

**Cochrane CENTRAL**

(osteocyte OR sclerostin OR Sost OR "osteocyte conditioned media" OR "osteocyte secretome" OR ("osteocyte" NEXT "extracellular" NEXT "vesicle") OR DMP1 OR Phex) AND ("brown adipose tissue" OR "brown fat" OR (beige NEXT adipocyte) OR browning OR thermogenesis OR UCP1 OR "uncoupling protein 1" OR PGC1A OR PRDM16 OR CIDEA OR DIO2 OR TMEM26)
